# Approaches towards Longevity: Reprogramming, Senolysis, and Improved Mitotic Competence as Anti-Aging Therapies

**DOI:** 10.3390/ijms20040938

**Published:** 2019-02-21

**Authors:** Sofia Melo Pereira, Rui Ribeiro, Elsa Logarinho

**Affiliations:** 1 Ageing and Aneuploidy Laboratory, IBMC, Instituto de Biologia Molecular e Celular, Universidade do Porto, 4200-135 Porto, Portugal; spereira@ibmc.up.pt (S.M.P.); rui.ribeiro@i3s.up.pt (R.R.); 2 i3S, Instituto de Investigação e Inovação em Saúde, Universidade do Porto, 4200-135 Porto, Portugal; 3 Cell Division Unit, Faculty of Medicine, Department of Experimental Biology, Universidade do Porto, 4200-319 Porto, Portugal

**Keywords:** aging, cellular senescence, cellular reprogramming, cell cycle fitness

## Abstract

Mainstream approaches that are currently used as anti-aging therapies primarily explore the senescence and epigenetic drift aging hallmarks and they are at two ends of the spectrum. While senolytic therapies include either the selective elimination of senescent cells or the disruption of their secretome with the use of drugs or natural compounds, cellular reprogramming uses genetic manipulation to revert cells all the way back to pluripotency. Here, we describe the progress that has been made on these therapies, while highlighting the major challenges involved. Moreover, based on recent findings elucidating the impact of mitotic shutdown and aneuploidy in cellular senescence, we discuss the modulation of mitotic competence as an alternative strategy to delay the hallmarks of aging. We propose that a regulated rise in mitotic competence of cells could circumvent certain limitations that are present in the senolytic and reprogramming approaches, by acting to decelerate senescence and possibly restore the epigenetic landscape.

## 1. Aging Hallmarks Explored in Anti-Aging Therapies: Epigenetics and Senescence

Aging is characterized by a progressive loss of physiological integrity and function over time [1]. Being the largest risk factor for the incidence of cancer, cardiovascular, and neurological diseases [2], it results from several interconnected molecular processes that decline with advancing age and that are commonly categorized in nine “aging hallmarks”: genomic instability, epigenetic alterations, telomere attrition, loss of proteostasis, deregulated nutrient sensing, mitochondrial dysfunction, cellular senescence, stem cell exhaustion, and altered intercellular communication [3]. Among these hallmarks, which are nevertheless interdependent, epigenetic alterations and cellular senescence have gained increased relevance, as they have been modulated by the current mainstream anti-aging therapies.

Aging epigenetics refers to changes in gene expression that naturally occur during an organismal lifespan without altering the DNA sequence. Chromatin epigenetics is regulated by several enzymes, which collectively give rise to modifications in DNA methylation and histone methylation/acetylation processes. During aging, the chromatin appears to be in a more active state, with an overall decrease in DNA methylation, thus leading to more relaxed global gene expression [4]. On the other hand, some studies have shown that several other chromatin sites, such as CpGs islands, bivalent chromatin domain promoters that are associated with key developmental genes, and Polycomb-group protein targets, are subject to age-related hypermethylation [5,6,7]. The analysis of DNA methylation (mDNA) status of specific CpGs islands allowed for the development of predictors of biological age, which are known as the “epigenetic clocks” [8,9,10,11,12]. Horvath’s multi-tissue age predictor is the most widely used with a correlation of 0.96 to chronological age and error margin of 3.6 years [10]. It has been established as one of the best age-predictor biomarkers [13], where the same epigenetic signature was found independently from cellular proliferative capacity or tissue-derived dynamics. In the case of the Hutchison–Gilford Progeria syndrome (HGPS), a severe laminopathy that is caused by a mutant form of Lamin A, known as progerin, an accelerated epigenetic aging was described in comparison to healthy individuals with the same chronological age [9]. Moreover, changes in chromatin localization can also drive histone alterations that occur during aging. For instance, nuclear lamins, which are the major components of the nuclear lamina and are responsible for maintaining nuclear shape, have been associated for upholding a repressive heterochromatin environment at the nuclear periphery [14]. Thus, in the HGPS laminopathy, there is a loss in epigenetic control of pericentric constitutive heterochromatin that is characterized by the down-regulation of heterochromatin mark, histone H3 trimethylated on lysine 9 (H3K9me3), and the up-regulation of histone H4 trimethylated on lysine 20 (H4K20me3). H3K9me3 reduction is accompanied by an overall reduction of its binding partner, heterochromatin protein 1α (HP1α) [15], and a decrease in their binding affinity. An increase of pericentric satellite III repeat transcripts is associated with this reduction, which suggests an up-regulation of the transcriptional activity of pericentric regions. Moreover, the up-regulation observed for H4K20me3, a mark for telomeric heterochromatin, blocks telomere elongation [16]. Overall, it appears to be consensual that epigenetic dysregulation is a key driver of aging and is thus responsible for the main alterations in gene expression within cells over time.

Another main driver of aging is cellular senescence. It refers to a state of permanent cell-cycle arrest that occurs in response to a variety of cell-intrinsic and -extrinsic stresses, such as nutrient deprivation, oncogenic activity, epigenetic stress, DNA damage, telomere shortening, and de-repression of the INK4/ARF locus, which irreversibly prevents the proliferation of damaged cells [17,18]. Senescence-inducing stressors usually engage either the p53-p21^CIP1^ or the p16^INK4A^-pRB tumor suppressor pathways, depending on stress or cell type, which can independently halt cell cycle progression [19]. DNA-damaging stressors activate p53 through DNA damage response (DDR) signaling (ionizing radiation [20,21], chemotherapeutics [22], oncogenic proliferation [23]), leading to the up-regulation of the p53 transcriptional target p21, which, in turn, induces a cell-cycle arrest by inhibiting cyclin E-Cdk2. p16^INK4A^ also inhibits cell-cycle progression, but tit does so by targeting cyclin D-Cdk4 and cyclin D-Cdk6 complexes. Both p21 and p16^INK4A^ then prevent the phosphorylation and inactivation of retinoblastoma (RB) [24,25,26], thus resulting in a steady repression of E2F-target genes that are required for cell cycle progression [17,27,28,29].

Although a single universal marker for cellular senescence is still to be unveiled, senescent cells present several distinguishing features in vitro, such as (i) flattened morphology and enlarged nuclear size [30]; (ii) increased senescence-associated β-galactosidase (SA-β-Gal) activity [31]; (iii) activation of p53 and p16^INK4A^-pRB tumor suppressor pathways that block cell cycle progression; (iv) activation of DNA damage response (53BP1 and γH2AX foci); and, (v) the formation of heterochromatin foci, enriched in chromatin modifications, such as S83-HP1γ, HIRA, ASF1, macroH2A, and H3K9me3, which remodel the transcriptional landscape [29,32,33]. Moreover, cellular senescence is accompanied by the development of a senescence-associated secretory phenotype (SASP), a distinctive cell-specific secretome that consists of various pro-inflammatory cytokines (e.g., IL-1α, -1β, -6, and -8), growth factors (e.g., VEGF, HGF, TGF-β, and GM-CSF), chemokines (e.g., CXCL-1, -3, and -10), serine proteases and their inhibitors (e.g., PAI-1), and matrix remodeling enzymes (e.g., MMP-1, -2, -3, -7, and -9) [34]. On a more extended view, SASP is not restricted to secreted bio-active factors, but it also includes membrane-bound cell surface ligands and receptors that exacerbate the cellular responses. For instance, the CXCR2 receptor binds to angiogenic CXC chemokine family members (including IL-8, CXCL-1, CXCL-3) that, by cooperating in an autocrine and paracrine fashion, reinforce senescence in a p53-dependent manner [35]. There has been intensive research examining the regulatory mechanisms behind cellular senescence and SASP. It is now clear that this occurs on two fronts; while p53 and pRB are responsible for halting cell cycle progression during cell senescence, the regulation of the secretory component seems to be mainly mediated by the NF-κB signaling pathway [36,37,38]. Recently, Hernandez-Segura and colleagues identified and validated a “core signature” of the senescence-associated transcriptome, based on RNA-sequencing datasets from melanocytes, keratinocytes, and astrocytes. This meta-analysis revealed a common signature of 55 genes that were strongly associated with specific stresses and cell types, enriched for gene ontology (GO) terms related with chromatin organization, DNA repair, and replication [39].

To this day, the biological significance of cellular senescence remains yet to be fully understood. If, on one hand, it works as a safeguard mechanism against tumorigenesis [30], accumulating evidence has suggested an impact on several biological processes, such as embryonic development [40,41], tissue repair/regeneration [42], inflammation [43], immunosurveillance [36,44,45,46], and angiogenesis [47]. However, the significance of cellular senescence as a driver of organismal aging has gained firm ground, with studies demonstrating that senescent cells contribute to an early onset of age-related phenotypes [48,49,50] and the accelerated progression of age-associated pathologies (diabetes, atherosclerosis, Alzheimer’s disease) [19]. The gradual accumulation of senescent cells coupled with the spread of senescence to neighboring healthy cells through a highly dynamic SASP, fuels a state of chronic sterile inflammation (indicating the absence of detectable pathogens) called “inflammaging” [43,51,52,53], thus entailing a deleterious spiral of increasing inflammation and the loss of tissue integrity and function. This dual role of cellular senescence is a clear example of evolutionary antagonistic pleiotropy, being beneficial at young ages but detrimental at older ages [54].

## 2. Aneuploidy as an Aging Hallmark Inter-Reliant with Senescence and Epigenetics

For several decades, many observations have demonstrated an incidence of aneuploidy along human chronological aging [55,56,57,58,59,60]. Aneuploidy is defined as an abnormal chromosome number resultant from chromosome mis-segregation during cell division, in both gametes and somatic cells. The molecular mechanisms behind the age-associated aneuploidy globally point to alterations in the expression levels of genes that are involved in the cell cycle and in the mitotic apparatus, which have been extensively reviewed in [61]. Interestingly, genomic instability, telomere erosion, epigenetic drift, and defective proteostasis, which are the primary hallmarks of aging acting as initiating triggers leading to secondary hallmarks, have all been reported to induce mitotic defects and aneuploidization [61]. Moreover, aneuploidy resulting from lagging chromosomes/weakened mitotic checkpoint has been associated with cellular senescence and premature aging in mice, reinforcing the potential role of aneuploidy in age-associated loss of tissue homeostasis [62,63,64,65]. Growing evidence has been showing aneuploidy to be able to drive the transition to a full senescent state, both in cases of mild [66] or highly [67,68] complex karyotypes that were observed in aged and cancer cells, respectively. Interestingly, micronuclei that were generated during defective mitoses can trigger an immunostimulatory response [69,70]. Such micronuclei occur after chromosome mis-segregation during cell division, consisting of chromatin surrounded by its own nuclear membrane. Micronuclear DNA is particularly susceptible to DNA damage, massive fragmentation, and leakage to the cytoplasm following spontaneous rupture of the micronuclear membrane [71,72]. The presence of cytosolic double-stranded DNA (dsDNA) activates the innate immune signaling, which elicits a pro-inflammatory response through the activation of the cyclic GMP-AMP synthase-stimulator of interferon genes (cGAS-STING) anti-viral pathway and the downstream interferon type I and NF-κB signaling pathways [73,74]. This results in cellular senescence and consequent SASP in an auto- and paracrine manner [69,70,75,76], which likely has implications in the cellular aging phenotypes.

Besides cellular senescence, aneuploidy has also been linked with epigenetic alterations, namely DNA hypomethylation. For instance, in acute lymphoblastic leukemia (ALL), most of the tri- and tetra-somic chromosomes were found to be significantly less methylated in gene-poor regions, when compared to their disomic autosomal counterparts [77]. Additionally, trisomies 7 and 14 in colon cancer have been reported to exhibit a decrease in DNA methylation [78]. In constitutional aneuploidy syndromes, such as Down syndrome, the extra chromosome 21 was shown to be responsible for the epigenetic changes that were observed in a *trans*-acting effect and mostly on other chromosomes [79]. More recently, aneuploidy was suggested as a direct cause of epigenetic instability in yeast with 3% of random aneuploid karyotypes disrupting the stable inheritance of silenced chromatin during cellular proliferation [80].

**Figure 1:**
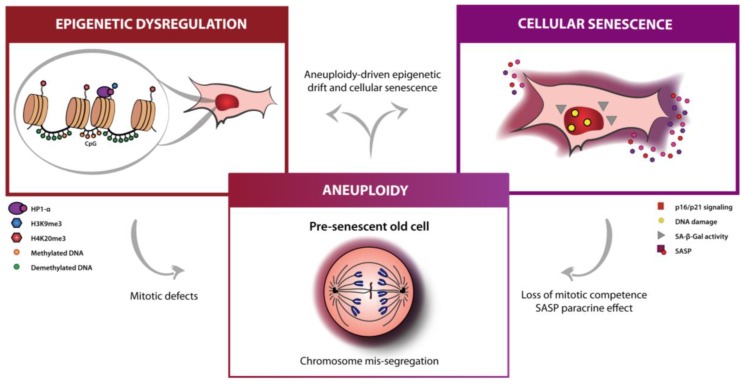
Epigenetic dysregulation, cellular senescence and aneuploidy: emerging targets for organismal rejuvenation and healthspan. Distinct changes in cells are observed during aging, including the accumulation of epigenetic alterations (global DNA demethylation and heterochromatinization, down-regulation of H3K9me3, up-regulation of H4K20me3, and delocalization of heterochromatin protein 1α (HP1-α)) and an evolving proinflammatory senescent phenotype (with activation of DNA damage and p16/p21 signaling pathways, senescence-associated β-galactosidase activity and of a highly secretory phenotype). An emerging hallmark, age-associated aneuploidy results from a gradual down-regulation of the mitotic machinery along aging, perhaps driven by the other hallmarks, but also shown to elicit epigenetic alterations and cellular senescence.

Altogether, the emergent findings point to age-associated aneuploidy as an aging hallmark that is caused by a steady down-regulation of the mitotic machinery over time. Even though aneuploidy is often categorized as a particular case of genomic instability, with evidence supporting that DNA damage and aneuploidy are inter-causal [81,82], still aneuploidy meets the criteria of an aging hallmark as (i) it manifests during normal aging [55,56,57,58,59,60], (ii) its experimental aggravation accelerates aging [62,63,64,65,66,67,68], with many aneuploidy-associated phenotypes being aging hallmarks [61], and (iii) its experimental inhibition delaying aging [66,83]. Importantly, aneuploidy is interconnected with both cellular senescence and epigenetic dysregulation, in one hand contributing to these other hallmarks, as discussed, but is also likely aggravated by them [61] (Figure 1). This raises interesting questions as to whether the effects of mainstream strategies targeting senescence and epigenetics act to delay aneuploidy and whether improvement of mitotic fidelity could act to prevent aneuploidy-driven senescence and epigenetic alterations during aging.

## 3. Mainstream Approaches for Anti-Aging Therapies: Partial Cellular Reprogramming and Senolysis

### 3.1. Partial Cellular Reprogramming

Yamanaka’s breakthrough on discovering that differentiated cells can be reverted to a pluripotent state by the expression of a small group of transcription factors (Oct4, Sox2, Klf4, and c-Myc, OSKM) opened the possibility that one day patient-specific cells could be transdifferentiated in vitro and was routinely used for cell replacement therapy [84]. The reversible nature of chromatin rearrangement with partial cellular reprogramming opens the exciting possibility of using therapeutic targeting of chromatin regulators to ameliorate aging hallmarks. However, the use of viral vectors raised safety issues that hampered the potential use of induced pluripotent stem cells (iPSCs) in regenerative medicine. In 2008, Yamanaka’s group reported the production of iPSCs with repeated transfection of expression plasmids [85] and, in 2009, two independent groups reported having achieved induced pluripotency from mouse and human fibroblasts, using OSKM proteins combined with cell-penetrating peptides [86,87]. A few years later, Hou and colleagues developed a new generation protocol to induce pluripotency in somatic cells using small-molecule compounds, designating it as chemically-induced reprogramming [88]. During the chemical induction of mouse fibroblasts, the cells undergo an extra-embryonic endoderm (XEN)-like intermediate state before reaching pluripotency, which is sufficient to directly reprogram cells into specific lineages and specified cell subtypes [89]. The methylation status of the DNA itself appears to be a crucial feature for effective reprogramming, as the correct DNA demethylation process was found to be highly important for the primordial stage of reprogramming, instead of the latter acquisition of pluripotency [90]. Interestingly, Vitamin C also has been shown to enhance the reprogramming efficiency [91] by promoting the activity of histone demethylases Jhdm1a and Jhdm1b [92]. The latter is responsible for accelerating cell cycle progression and suppressing cell senescence during the reprogramming process [92]. All of these advances in the last years might allow for standardizing safe, controllable, and clinically relevant protocols for specific cell type production, without having to revert differentiated cells to their pluripotency state, thereby surpassing the risks of tumorigenesis.

In the context of premature aging, iPSCs that were derived from fibroblasts of HGPS patients and healthy donors were reported to be nearly indistinguishable in terms of pluripotency, nuclear architecture, transcriptional, and epigenetic profiles [93]. However, once allowed to differentiate into vascular smooth muscle cells, HGPS cells recapitulated disease progression, with progerin expression starting shortly after differentiation, accompanied by premature aging phenotypes, such as decreased proliferation, premature loss of peripheral heterochromatin, increase in senescence, and decreased telomere length [93,94]. Despite reprogramming-induced reset of epigenetic dysfunction, cellular senescence [94], transcriptome landscape, telomere size, oxidative stress, and mitochondrial metabolism [95], not all aging-associated hallmarks can be reset. For instance, the accumulation of nuclear and mitochondrial DNA damage is an aspect of aging that might not be rescued by reprogramming technology [96]. Nevertheless, rejuvenation during organismal aging may be possible if partial reprogramming is continued over time. In 2016, Ocampo et al. induced partial reprogramming by the cyclic expression scheme of OSKM transcription factors in a premature aging HGPS mouse model. Tightly controlled transient reprogramming of cells over time allowed for the cells to enter an intermediate state, inducing cellular rejuvenation without the loss of cellular identity and function. The authors reported not only improving cellular and physiological hallmarks of aging but also prolonging lifespan [97]. A similar approach was performed *in vitro*, with “interrupted reprogramming” in murine epithelial cells as a strategy to generate induced progenitor-like cells, allowing for controlled expansion but maintaining the ability to efficiently return to their original phenotype [98]. Arguably, in the case of mesenchymal stromal cells, the interruption of in vitro reprogramming process before cells reach pluripotency, did not prolong cell expansion or improve the molecular and epigenetic hallmarks of senescence. Moreover, the continued transfection of the OSKM factors promoted cell transformation [99]. Still, when full pluripotency is achieved in vivo by the forced expression of OSKM factors, it results in tumor development in multiple organs due to altered epigenetic regulation [100], with the acquisition of totipotency, expressing markers for both embryonic and extra-embryonic layers, which are normally absent in standard iPSC or ES cells [101]. A recent study has analyzed the dynamics of epigenetic aging during human iPSC reprogramming and found that partial reprogramming leads to a steady reduction in the epigenetic age, proportional to the loss of somatic gene expression [102]. The TRA-1-60 (+) cells were reported as partially reprogrammed between day 7 and 11 after OSKM transduction, presenting the high expression of pluripotency genes, accompanied by a high reversion rate towards the somatic state [103]. Oppositely, in Ocampo et al., partial reprogramming was achieved in only two days in mice carrying a doxycycline-inducible OSKM transgene system [97]. It has been shown that transgenic mice carrying an inducible OSKM transgene have a 25–50-fold greater efficiency than observed using direct transduction with a lentiviral system under the same inducible promoter [104]. Nevertheless, using the epigenetic clocks that are available to measure epigenetic age during iPSC reprogramming, it appears that the rejuvenation process occurs within the uncommitted reprogramming phase before cells lose their somatic identity [102]. Overall, the cell reprogramming technology appears to be a highly valuable resource as an anti-aging strategy, suggesting that partial epigenetic reprogramming can indeed be used as a rejuvenation mechanism in human cells.

### 3.2. Senolytic Therapies and Clearance of Senescent Cells

Aging is a biological process that can be positively or negatively influenced by environmental factors. Many natural antioxidants are known for long to promote a positive influence on aging kinetics, such as vitamin E [105], kinetin [106], carnosine [107], and garlic [108]. Some noteworthy examples of anti-aging drug discovery are the use of small molecules, such as resveratrol, targeting the pharmacological manipulation of sirtuin 1 [109], rapamycin, through the inhibition of the mTOR signaling pathway [110], and metformin, which was initially approved as a drug to treat diabetes, but that appears to target a number of aging-related mechanisms [111]. More recently, clearance of senescent cells has been targeted as a potential anti-aging therapy, with focus on alleviating or delaying age-related diseases. A few distinct classes of compounds with evidence of senolytic properties have been identified: natural compounds (such as quercetin [112], piperlongumine [113], and fisetin [114]), BCL2 family inhibitors [115,116], forkhead box protein O4 (FOXO4)-interacting peptide [117], Hsp90 inhibitors [118], and histone deacetylase inhibitor [119]. These compounds have been reviewed in [120,121].

To support the hypothesis that senescent cells are responsible for the predisposition of age-related dysfunction, small numbers of senescent cells were introduced into young (six-month old) mice and this was enough to cause physical dysfunction, earlier occurrence of age-related diseases, and shorter survival. An intermittent pharmacological intervention with the combination of dasatinib plus quercetin, in both senescent cell-transplanted young mice and normally aged mice, improved overall fitness and survival [122]. This senolytic cocktail has also been found to be effective as an alternative method for the treatment of age-related osteoporosis [123] and of hepatic steatosis [124]. As a long-term pharmacological treatment, it was also found to improve the vasomotor function in established aging-associated vascular phenotypes and in chronic hypercholesterolemia [125]. The exact mechanism of action dasatinib plus quercetin on senescent cells remains unknown. Emerging evidence suggests that, apart from senescent cell clearance, there is a recovery of cell proliferative capacity. For instance, quercetin is a widespread flavonoid that is derived from plants and it has been extensively reported to have strong anti-inflammatory and immune-enhancement capacity [126]. When used at low dose (100 nM), it was found to effectively reduce senescence levels by increasing cell proliferation and restoring the heterochromatin architecture in human mesenchymal stem cells (hMSCs) of the Werner syndrome (WS) premature aging model. In here, the percentage of senescent cells was significantly reduced, which was primarily due to the proliferative capacity rescue of surrounding cells, as shown by a 2.5-fold increase in Ki67 staining and a consequent overall decrease of p16 and p21 markers, DNA damage-response markers γ-H2AX and 53BP1, reactive oxygen species (ROS) production, mRNA levels of proinflammatory cytokine IL-6, and cell apoptosis. Also, in HGPS, quercetin alleviated the cellular senescence and physiological aging of hMSCs by reducing the progerin levels, decreasing population doubling time, decreased SA-β-Gal percentage, and increased clonal expansion and proliferative ability. Moreover, transcriptome analysis revealed that quercetin improved WS-hMSC through the up-regulation of genes that are involved in cell cycle, cell division, chromosome segregation, and cell proliferation [127]. Comparatively, vitamin C was also found to improve the aging defects in WS-hMSCs. Once more, RNA sequencing revealed that the vitamin C mode of action altered the expression of several genes that are involved in chromatin condensation, cell cycle regulation, DNA replication, and DNA damage repair pathways [128]. When quercetin and vitamin C transcriptional profiles were compared, 153 up-regulated genes were found to be common in both treatments, enriched in biological processes GO terms related to cell cycle, chromatin condensation, and anti-oxidation [127]. Additionally, in a different report, 0.1 mM of vitamin C was found enough to significantly increase the in vitro proliferative capacity of bone-resident osteoblasts and decrease the amount in senescent cells. Likewise, transcriptome analysis revealed that, in the presence of vitamin C, the main pathways activated are apoptotic, cell cycle-proliferation, and catabolic pathways [129]. Altogether, senolytic therapies aim at the selective clearance of senescent cells with the ultimate goal to delay age-related disorders by keeping tissues and organs cleared of senescent cells and their distinctive pro-inflammatory phenotype. However, the question of whether these compounds effectively clear senescent cells or act either on the microenvironment or on the proliferating capacity of healthy cells, consequently diluting the senescent cell population (and thus the overall secretory phenotype) remains unanswered.

## 4. Modulation of Mitotic Competence as a New Anti-Aging Therapy

Despite being widely accepted, why is cell proliferative capacity lost during aging, why do cell cycles slow down and become erroneous with age, and what ultimately causes elderly cells to stop dividing has remained unclear. An intriguing link between loss of mitotic fidelity and aging was originally evidenced by van Deursen and co-workers while studying a series of mice with a graded reduction in the expression of the spindle assembly checkpoint protein BubR1 [62]. Despite developing progressive aneuploidy, these mice did not present increasing spontaneous tumorigenesis. Instead, they exhibited a variety of premature aging features (e.g., shortened lifespan, growth retardation, sarcopenia, cataracts, loss of subcutaneous fat, and impaired wound healing) and premature cellular senescence [62]. Remarkably, sustained overexpression of BubR1 was later shown to extend lifespan and delay age-related degeneration and aneuploidy [83]. These findings, combined with the observation that BubR1 expression, declines drastically in aged mouse tissues [62] has suggested that BubR1 functions as a key regulator of the natural aging process, through its role on the mitotic checkpoint. Consistent with the idea that aneuploidy drives the loss of tissue homeostasis, double-haploinsufficient mice for the mitotic checkpoint proteins Bub3 and the Bub3-related protein Rae1 [130] were also shown to exhibit early traits of aging [63]. Nonetheless, not every aneuploidy-prone mouse model is reported to develop traits of premature aging. At first sight, this seems to argue against the idea that aneuploidy can drive aging. This might be explained due to the fact that (i) most of these chromosomally-unstable mouse models present high aneuploidy rates, leading to tumorigenic development and a premature sacrifice before the onset of the aging phenotypes; (ii) the onset of the aging phenotype might require synergistic action from other cellular stressors, which engage senescence pathways; and, (iii) genes that trigger chromosome instability (CIN) when mutated, causing aneuploidy [131], might be organized in a hierarchy playing multiple functions outside cell division.

The role of CIN/aneuploidy in the process of chronological aging is yet to be fully disclosed. Though informative, the BubR1-deficiency model suffers the drawback that the induced aneuploidy rates are non-physiological and they are present from early development onwards, not resembling the steady accumulation of aneuploid cells that will senesce during aging. Further, the impact of senescent cells seems to depend on their gradual accumulation on the tissue to give rise to an increasing inflammatory loop. BubR1-hypomorphic mouse embryonic fibroblasts senesce very quickly and in a much higher extent than the naturally aged BubR1-proficient counterparts [50]. Progress in the understanding of how senescent cells arise during aging appears to rely on the identification of the precise molecular mechanism underlying age-associated chromosomal instability. The first steps towards this identification were taken by the work of Ly and colleagues, which suggested mitotic dysfunction as a driver for chromosomal pathologies during aging, through a comparative analysis of gene expression in natural and accelerated aging human samples [132]. Although a pioneer, this study based on mixed populations of cells at different stages of the cell cycle limited the conclusions regarding mitotic gene repression in elderly cell cultures with a lower mitotic index. Recently, our group tackled this caveat by focusing on purified mitotic subpopulations [66]. Through direct long-term live-cell imaging of young, middle-aged, and old-aged primary human dermal fibroblasts, we found an increased frequency of mitotic abnormalities in older cells, resulting in mild aneuploidy levels. RNA-sequencing analysis of mitotic extracts of young and old cells further confirmed a transcriptional shutdown of the mitotic gene cluster, with the Forkhead box M1 (FoxM1) transcription factor being disclosed as the molecular determinant of this age-associated mitotic decline. FoxM1 is the main driver of the G2/M transition transcriptional program in mammalian cells [133]. Being tightly correlated with the cell’s proliferative rate, FoxM1 is expressed in all embryonic tissues, but is then restricted to adult tissues with a high proliferation index [134]. FoxM1 expression can also be induced following tissue injury, as shown for regenerating livers after both chemical insult [135,136] and partial hepatectomy [137]. The RNA-sequencing datasets also revealed an up-regulation of the senescence-core signature and SASP gene clusters that correlated with this dysfunction on mitotic machinery [39], indicating an unforeseen senescent phenotype in elderly diving cells. We further questioned whether the mild aneuploidy levels occurring during aging also account for the accumulation of senescent cells. Through innovative experimental layouts, such as aneuploidy measurement in fluorescence-activated cell-sorted senescent cells and long-term live-cell microscopy, we demonstrated that the FoxM1-depleted aneuploid cells ultimately engage a permanent cell cycle arrest, evolving into full-blown cellular senescence and a highly active SASP. Conversely, reinstating the FoxM1 transcriptional activity of old cells to levels of young cells rescued the loss of mitotic proficiency and delayed cellular senescence. Altogether, the data gathered in this work support a model in which proliferating naturally aged cells undergo a FoxM1-driven mitotic shutdown, with simultaneous senescence-associated gene expression signature (early senescence state). Elderly cells ultimately generate aneuploid progeny, which significantly accounts for the accumulation of the full senescent phenotype (permanent cell cycle arrest and SASP), ultimately driving aging. Noteworthy, several studies have also demonstrated a positive correlation between increased FoxM1 levels and poor cancer prognosis endowing cancer cells with over-proliferative capacities [138]. However, clear evidence of FoxM1-induced tumorigenesis in a homeostatic setup is still missing, as it has only been shown to have tumorigenic potential in combination with oncogenic mutations [138,139,140,141].

**Figure 2:**
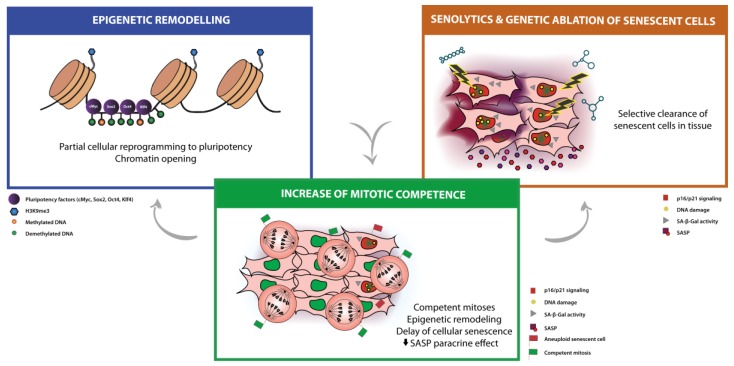
Epigenetic reprogramming, senolysis and modulation of mitotic competence: emerging strategies for organismal rejuvenation and healthspan. Epigenetic reprogramming and selective clearance of senescent cells are already being explored in the bench as anti-aging approaches. Modulation of mitotic fitness emerges as a new potential strategy to take into consideration as anti-aging therapy, by allowing the reversion of the dysregulated epigenetic landscape and delaying the accumulation of senescent cells and senescence-associated secretory phenotype (SASP)-induced inflammatory microenvironment.

With the emergence of aneuploidy as a candidate hallmark of aging, these intriguing findings opened a new window regarding the biology of aging, by suggesting an unexpected positive feedback loop between cellular aging and aneuploidy that can be further explored. A recent study strengthens this new concept of restoring the cells’ proliferative capacity as a means of preventing cellular senescence and the auto- and paracrine inflammatory loops of SASP [142]. Here, Bussian et al. established a direct link between the accumulation of senescent astrocytes and microglia, the proliferative cell populations in the brain, and cognition-associated neuronal loss in a mouse model of tau-dependent neurodegenerative disease. The continuous clearance of p16-positive astrocytes and microglia using the *INK-ATTAC* “suicide” transgenic approach, before disease onset, had a profound positive effect on disease progression, preventing gliosis, neurofibrillary tangle formation, neurodegeneration, and cognitive decline. The clearance of senescent cells using the senolytic compound ABT263 (navitoclax) had similar effects, reducing the accumulation of neuronal tau phosphorylation, thus preventing its aggregation [142]. Overall, it appears to be crucial that the proliferative capacity of astrocytes and microglia is not hampered for proper brain function. Nevertheless, these new data highlight the impact of senescence acquired by proliferative cell types in the healthy status of neighboring differentiated cells in the tissue, supporting the modulation of mitotic competence and fidelity as a promising anti-aging strategy to counteract cellular senescence (Figure 2 and Table 1).

## 5. Concluding Remarks and Future Directions

Nowadays, there is a rapidly increasing trend for aging populations, which will translate into a significant burden in healthcare systems. The reversible nature of chromatin rearrangement with partial cellular reprogramming opens the exciting possibility of using therapeutic targeting of chromatin regulators to rescue the aging hallmarks. The concept that cellular differentiation is a bidirectional process, and that cell fate is flexible through partial cellular reprogramming, is very appealing for future patient-derived cell replacement therapies. It appears that we are now facing the beginning of the rejuvenation era, with epigenetics considered by many of the most conserved aging hallmarks [144,145], and the know-how in precise epigenetic modulation expected to disclose standardized rejuvenation platforms that will improve healthspan. On the other hand, several reports point to the accumulation of senescent cells in tissues and organs as having a significant impact on age-related pathologies, with the selective clearance of these cells leading to a healthier and longer life [48,146]. Even a relatively small percentage of senescence in an organism, as 10–15% described for aged primates, is enough to cause a significant decline in physiological function [147]. Senolytic therapies drive the hypothesis that targeting a fundamental key factor in the aging process, such as cellular senescence, will delay age-related diseases as a group, instead of a single disease in detriment of another. We are left to learn more what a truly senescent cell is, if there is the need of long- or short-term clearance from the organism, and, more importantly, if we can rescue the still proliferative “pre-senescent” cells. In this context, a new candidate hallmark for aging arises, aneuploidy, an abnormal chromosomal number that results from mis-segregation events during mitosis, which has been linked to normative aging and age-associated diseases, with the underlying mechanisms being poorly understood. Recently, aneuploidy was shown to increase with advancing age due to an overall dysfunction of the mitotic machinery [66]. Furthermore, several reports have uncovered the impact of aneuploidy on cellular fitness and proliferative capacity [148,149,150], with several characteristics of aneuploid cells overlapping with those that are found in aged cells. Interestingly, as mitosis slows down with advancing age, so does the cumulative rate of mitotic defects. Mitotic decline that is observed during aging is primarily due to the repression of the FoxM1 transcription factor that drives the expression of the late cell cycle gene cluster. The accumulation of macromolecular damage, including DNA damage, accounts for FoxM1 repression. It would be interesting to determine whether the current mainstream anti-aging therapies modulating epigenetics and clearing senescence impact FoxM1 expression. As discussed, emerging evidence support that both cellular reprogramming [92] and senolytic compounds [127,128,129] up-regulate the expression of genes that are involved in cell cycle progression and cell division fidelity.

Although, it is well accepted that mitotic competence is largely affected by epigenetic alterations [45,61,151,152,153] and senescence [154,155,156,157,158], the inverse link is now being considered. In fact, we previously found that the restoration of mitotic competence through the re-establishment of FoxM1 expression in elderly and HGPS fibroblasts is able to alleviate senescence phenotypes and SASP and our RNA-sequencing profiling disclosed a FoxM1-driven modulation of several epigenetic regulators, such as HP1α, SUV39H1, and HDAC1 [66]. Evidence of accelerated epigenetic aging in trisomy 21 patients [159] and of low levels of cellular senescence in BubR1-overexpressing mice [83] reinforce the impact of mitotic competence in the epigenetics and cellular senescence. What now remains to be fully understood is whether this is through the inhibition of aneuploidy or effects in other cellular non-mitotic processes. Nevertheless, aneuploidy has been associated with the activation of innate immune signaling [69,70] and epigenetic alterations [80], supporting that it might be a good therapeutic target in the context of aging. Our latest work provided insight as to how senescent cells arise, by demonstrating that elderly proliferative cells, primed with the expression of a senescence core gene signature, evolved into permanent cell cycle arrest (full senescence) following passage through a faulty mitosis [66]. This further supports that improving mitotic fitness may be used as a potential anti-aging strategy, thereby counteracting the SASP-induced inflammatory microenvironment and helping to protect the stem cell and parenchymal cell functions. Indeed, recent findings have shown the selective targeting of senescent astrocytes and microglia (proliferative cells in the brain) to successfully revert tau protein aggregation in neurons. By improving the mitotic fitness, one could prevent the deleterious accumulation of aneuploid senescent cells, preserving the cellular and tissue homeostasis, and impacting the organismal healthspan. Thus, in order to further test this idea, it will be important to optimize the detection and quantification of aneuploid senescent cells in vivo and investigate the molecular mechanisms by which these cells influence stem and parenchymal cell aging.

## Figures and Tables

**Table 1:** Studies reporting aging therapeutic/preventive strategies that show improvement of cell proliferative fitness.

Study	Therapeutic/Preventive Rejuvenation Strategy	Epigenetic Modulation	Decrease in Cellular Senescence	SASP Modulation	Improvement of Cell Proliferative Fitness	Ref.
	**Reprogramming**					
Esteban 2010	Vitamin C promoted generation of mouse and human iPSCs		√		√	[91]
Wang 2011	Histone demethylases Jhdm1a/1b identified as key effectors in vitamin C induced reprogramming	√	√		√	[92]
Liu 2011	Reprogramming of HGPS cells alleviated progeroid phenotypes	√	√		√	[94]
Ocampo 2016	Transient expression of OSKM factors alleviated age-associated symptoms, prolonged lifespan in progeroid mice and improved tissue homeostasis in older mice	√	√	√	√	[97]
	**Senolysis**					
Baker 2011	Long-life and late-life ablation of p16-positive cells delayed or attenuated progression of age-related disorders		√ ^2^	√	√	[48]
Jeon 2017	Ablation of p16-positive cells/ use of senolytic compound UBX0101 attenuated the development of post-traumatic osteoarthritis and created a pro-regenerative environment		√ ^2^	√	√	[143]
Xu 2018	Combination of Quercetin + Dasatinib extended both health- and lifespan in aged mice		√	√	√ ^1^	[122]
Geng 2018	Quercetin rejuvenated WS, HGPS and chronologically-aged hMSCs	√	√	√	√	[127]
Li 2016	Vitamin C rejuvenated WS hMSCs	√	√	√	√	[128]
Burger 2017	Vitamin C attenuated senescence of human osteoarthritic osteoblasts		√		√	[129]
Chang 2016	ABT263-induced senescent cell clearance and rejuvenated aged hematopoietic stem cells (HSCs) and muscle stem cells (MuSCs)		√ ^2^	√	√	[116]
Fuhrmann-Stroissnigg 2017	HSP90 inhibitor 17-DMAG delayed onset of age-associated symptoms in a progeroid mouse model		√ ^2^	√	√	[118]
	**Mitotic Competence**					
Baker 2012	High-level expression of BubR1 extended lifespan and delayed age-related deterioration and aneuploidy in several tissues		√		√	[83]
Macedo 2018	Restoring levels of FoxM1 in elderly and HGPS cells reestablished mitotic proficiency and reduced senescence	√	√	√	√	[66]

^1^ Not statistically significant. ^2^ Selective clearance of senescent cells.

## Abbreviations

mDNA	DNA methylation
HGPS	Hutchison-Gilford Progeria syndrome
H3K9me3	H3 trimethylated on lysine 9
H4K20me3	H4 trimethylated on lysine 20
HP1α	Heterochromatin protein 1α
DDR	DNA damage response
RB	Retinoblastoma
SA-β-Gal	Senescence-associated β-galactosidase
SASP	Senescence-associated secretory phenotype
GO	Gene ontology
dsDNA	Double-stranded DNA
cGAS-STING	Cyclic GMP-AMP synthase-stimulator of interferon genes
ALL	Acute lymphoblastic leukemia
iPSC	Induced pluripotent stem cells
XEN	Extra-embryonic endoderm
OSKM	Oct4, Sox2, Klf4, c-Myc
FOXO4	Forkhead box protein O4
hMSCs	Human mesenchymal stem cells
WS	Werner syndrome
ROS	Reactive oxygen species
CIN	Chromosomal instability
FoxM1	Forkhead box M1
HSCs	Hematopoietic stem cells
MuSCs	Muscle stem cells
